# Preparation of the endometrium for frozen embryo transfer: an update on clinical practices

**DOI:** 10.1186/s12958-023-01106-5

**Published:** 2023-06-08

**Authors:** Yiting Zhang, Xiao Fu, Shuli Gao, Shuzhe Gao, Shanshan Gao, Jinlong Ma, Zi-Jiang Chen

**Affiliations:** 1 Center for Reproductive Medicine, the Second Hospital, Cheeloo College of Medicine, Shandong University, Jinan, 250012 Shandong China; 2 Key laboratory of Reproductive Endocrinology of Ministry of Education, Shandong University, Jinan, 250012 Shandong China; 3 Shandong Key Laboratory of Reproductive Medicine, Jinan, 250012 Shandong China; 4 Shandong Provincial Clinical Research Center for Reproductive Health, Jinan, 250012 Shandong China; 5 Shandong Technology Innovation Center for Reproductive Health, Jinan, 250012 Shandong China; 6 National Research Center for Assisted Reproductive Technology and Reproductive Genetics, Shandong University, Jinan, 250012 Shandong China; 7 Shanghai Key Laboratory for Assisted Reproduction and Reproductive Genetics, Shanghai, 200135 China; 8 Center for Reproductive Medicine, Ren Ji Hospital, School of Medicine, Shanghai Jiao Tong University, Shanghai, 200135 China

**Keywords:** Frozen–thawed embryo transfer, Endometrial preparation, Hormone replacement therapy, Natural cycle, Luteal phase support, Endocrine monitoring, Endometrial assessment

## Abstract

Over the past decade, the application of frozen-thawed embryo transfer treatment cycles has increased substantially. Hormone replacement therapy and the natural cycle are two popular methods for preparing the endometrium. Hormone replacement therapy is now used at the discretion of the doctors because it is easy to coordinate the timing of embryo thawing and transfer with the schedules of the in-vitro fertilization lab, the treating doctors, and the patient. However, current results suggest that establishing a pregnancy in the absence of a corpus luteum as a result of anovulation may pose significant maternal and fetal risks. Therefore, a ‘back to nature’ approach that advocates an expanded use of natural cycle FET in ovulatory women has been suggested. Currently, there is increasing interest in how the method of endometrial preparation may influence frozen embryo transfer outcomes specifically, especially when it comes to details such as different types of ovulation monitoring and different luteal support in natural cycles, and the ideal exogenous hormone administration route as well as the endocrine monitoring in hormone replacement cycles. In addition to improving implantation rates and ensuring the safety of the fetus, addressing these points will allow for individualized endometrial preparation, also as few cycles as possible would be canceled.

## Background

In recent years, frozen–thawed embryo transfer (FET) technology has become mainstream because of the rapid development of assisted reproductive technology (ART) and the continuous progress of vitrification technology. The use of “freeze-all’’ strategy with subsequent FET is a promising option to reduce the iatrogenic risk of ovarian hyperstimulation syndrome (OHSS), perform pre-implantation genetic testing, avoid embryo-endometrial asynchrony in fresh cycles, and achieve a high live birth rate (LBR) and reliable safety [1–4]. Endometrial preparation is a crucial stage in FET cycles, and several protocols are available, including true natural cycle with spontaneous ovulation, modified natural cycle with human chorionic gonadotrophin (hCG) to trigger ovulation, hormone replacement therapy (HRT) cycle with or without gonadotropin-releasing hormone agonist (GnRH-a) downregulation, and ovarian stimulation cycle with or without letrozole. However, there is no consensus on the optimal endometrial preparation protocol for FET despite almost four decades since the first successful pregnancy after an FET cycle [5].

Although studies showed that FET significantly improves clinical outcomes and allows consecutive embryo transfers, it also has a higher risk of pregnancy-related hypertensive disorders, post-term delivery, macrosomia, and other adverse obstetrical or prenatal outcomes, especially for HRT cycles [6]. As a result, some scholars have suggested a “back to nature” approach, advocating for expanded use of natural cycle FET. Therefore, it is imperative to have a more scientific basis for FET administration due to the rising number of FET procedures being performed around the world.

This systematic review aims to provide up-to-date information on the reproductive, obstetric, and maternal outcomes of the two most commonly used endometrial preparation methods (the natural cycle and the HRT cycle with or without GnRH-a), as well as to discuss any contentious aspects that have arisen in clinics in recent years.

## Materials and methods

This review will discuss the natural cycle and hormone replacement therapy (HRT) cycle prior to frozen embryo transfer (FET), including their advantages, disadvantages, and effects on implantation and pregnancy outcomes. A literature retrieval was conducted on the PubMed and EMBASE databases for studies using the keywords “endometrial preparation,“ “frozen embryo transfer,“ “frozen–thawed embryo transfer,“ “natural cycle,“ “modified natural cycle,“ “hormone replacement treatment cycle,“ “hormone replacement treatment cycle transfer with gonadotropin downregulation,“ and MeSH terms “cryopreservation and pregnancy.“ All eligible articles published until November 2022 were thoroughly reviewed.

### Main text

#### Hormone replacement treatment (HRT)

In the hormone replacement therapy (HRT) cycle, exogenous estrogen supplements are used to stimulate the growth of the endometrium and inhibit follicular growth. The HRT cycle is more flexible and convenient, making it easier to schedule transplantation and resulting in a lower cancellation rate compared to the natural cycle. This is why some clinicians prefer the HRT cycle. However, the main drawbacks of this method are the potential adverse risks caused by exogenous estrogen supplementation and the absence of corpus luteum.

#### Estrogen Administration

Estradiol may be administered either as a fixed dose of 6 mg or in step-up regimens starting at 2 mg and increasing to 6 mg over 10 to 15 days. The fixed-dose regimen aims to prevent follicular growth and ovulation escape, while step-up regimens aim to increase estradiol exposure in a more physiologic manner. A large retrospective cohort study reported no difference in live birth rates (LBRs) between the fixed dose and step-up dose of estradiol after fresh embryo transfer (ET) in oocyte donation cycles with oral or transdermal supplementation [7]. This conclusion was supported by another recent retrospective study [8]. However, a recent single-center retrospective study using only transdermal patches as estrogen replacement [9], found that the constant dose regimen was associated with comparable LBR and obstetric outcomes but with a lower spontaneous abortion rate than that observed in the increasing regimen. In addition, a recent retrospective cohort study compared step-up from 2 mg, step-up from 4 mg, and fixed-dose 6-mg estrogen replacement regimens in hormone replacement therapy-frozen embryo transfer cycles. The study reported that a step-up regimen starting from 4 mg resulted in a significantly thicker endometrium and a tendency for higher clinical pregnancy and LBRs [10]. The author speculated that in the step-up from 4 mg group, enough estrogen priming of the endometrium leads to endometrial growth and the production of enough progesterone receptors to allow progesterone stimulation to achieve endometrial receptivity. Based on the above research, both a constant dose regimen and a step-up regimen from 4 mg could achieve satisfactory outcomes.

In terms of the lower limit of the duration of estradiol, 5–7 days may suffice for adequate endometrial priming [11, 12], but there is a need to be cautious about the occurrence of early abortion. Regarding the upper limit for the duration of estradiol priming, the length of estradiol treatment can be as long as 4 weeks without a negative impact on the LBR, but fetal birth weight and Z-score decreased with prolonged estrogen exposure when estrogen was administered for more than 36 days [13]. Therefore, we conclude that when endometrial thickness is appropriate, clinicians can be flexible in scheduling FET procedures without being limited by the number of days of estrogen administration, but it takes careful planning and organization to maintain optimal pregnancy rates and mother-infant safety.

Estrogen can be given as an oral or a vaginal tablet, a transdermal patch, and a subcutaneous or intramuscular injection. For oral administration, estradiol is easily converted to estrone, with steady-state estrone levels around 3–6 times higher than those of estradiol [14, 15]. Transdermal, intramuscular, or vaginal parenteral administration, on the other hand, can avoid first-pass hepatic metabolism, resulting in significantly less absorbed estradiol, which yields the most steady-state levels of estradiol and has been proposed to be preferred over the oral route for induction of endometrial receptivity [16]. Krasnow [16] concluded that the endometrial glandular histology in the oral protocol was delayed by an average of 1.6 days compared to that among women given transdermal estradiol. But in two randomized controlled trials (RCTs) [17, 18], there was no significant difference between transdermal estradiol and oral estradiol in the thickness of the endometrium on the day of progesterone administration or in the clinical outcomes. However, they both provided enough evidence that estradiol transdermal patches can be used instead of oral estradiol in FET cycles due to the reduced costs, drug dosage, and emotional stress, as well as the simplicity of the protocol for patients. In terms of various parenteral routes, vaginal or transdermal methods are frequently employed in many reproductive centers. A prospective monocentric cohort study reported no difference in clinical pregnancy rates between the two routes, but transdermal estrogen was associated with higher endometrial thickness, shorter treatment duration, fewer side effects, higher patient satisfaction, and lower levels of serum estradiol concentration in artificial FET cycles compared to the vaginal route [19]. In terms of perinatal outcomes, there was only one retrospective monocentric cohort on this topic [20]. It showed that the birth weights and perinatal complications were comparable when estradiol was administered transdermally or vaginally. However, the vaginal route is not generally preferred because it can cause local vaginal discomfort, irritation, and poor absorption, particularly when combined with vaginal progesterone. Also, the vaginal route is usually adopted as a supplement to oral or transdermal estradiol instead of as a starting administration route, due to the extremely higher concentrations of estradiol in serum and endometrium than the other two routes.

#### Progesterone administration

Regarding progesterone (P) supplementation itself, there is little agreement on the ideal route of administration and dose **(**Table 1**)**. Multiple routes of P administration are available, including oral, intramuscular (IM), vaginal, and a recently developed subcutaneous (SC) preparation. However, the efficacy, safety, and tolerability of exogenous P depend on the route of administration. Vaginal and IM are the preferred routes, but most patients prefer vaginal over IM P administration for greater convenience, ease of use, and less pain [21]. Recently, there have been a growing number of studies comparing the vaginal route with the IM route, but no consistent conclusions have been reached. Retrospective data are conflicting, being in favor of the IM route [22, 23] or showing no significant differences in terms of outcomes [24, 25]. Devine et al.[26] randomized 645 FET cycles into three treatment groups: 200 mg vaginal tablet P twice daily, 50 mg daily IM P, and vaginal P twice daily supplemented with 50 mg IM P every third day. The miscarriage rate was higher in the vaginal P-only group, which led to considerably lower ongoing pregnancy rates (31% vs. 47% and 50%; P < 0.0001) than in the other two groups. As a result, patient recruitment for the other two groups continued while the vaginal P-only group was prematurely stopped in the meantime. In the final analyses [27], there was no significant difference between IM P and vaginal P supplemented with IM P every third day. However, the LBR was considerably lower in the vaginal P-only arm (27%) compared to IM P (44%) and vaginal P supplemented with IM P every third day (46%). To date, however, these results have not been replicated in other RCTs [28, 29]. The typical IM P dose ranges from 50 to 100 mg/day, while vaginal P doses vary widely, from vaginal gel 90 mg once or twice daily or vaginal P tablets 100 mg two or three times a day to micronized P capsules 200 mg, 3–4 times a day, and vaginal pessaries 400 mg twice a day [22–24]. On the other hand, relatively little information comparing the pregnancy outcomes of various vaginal routes in planned FET cycles has been reported [30].

**Table 1:** Characteristics of the studies included in the systematic review of progesterone supplementation in HRT-FET

Source	Design	Sample size	Progesterone route	Progesterone dose corresponding to route	Day of embryo transfer	Outcomes
Wang, Y., et al. (2015)	RCT	1500	VG P(gel) + oral DYD IM P + D	90 mg/d + 20 mg/d 40 mg/d + 20 mg/d	Day 3	n.s.
Devine, et al. (2018) Interim analysis	RCT	645	VG P(tablet) vs. IM P vs. VG (tablet) + IM P	200 mg/d 50 mg/d 200 mg×2 /d + 50 mg/e 3rd day	Day 5 afternoon Day 5 afternoon Day 6 afternoon	OPR; P＜0.0001 31% vs. 47% vs. 50%;
Devine, et al. (2021) Final analysis	RCT	1060	VG P(tablet) vs. IM P vs. VG (tablet) + IM P	200 mg/d 50 mg/d 200 mg×2 /d + 50 mg/e 3rd day	Day 5 afternoon Day 5 afternoon Day 6 afternoon	LBR; P＜0.0001 27% vs. 44% vs. 46%;
Jiang, L., et al. (2019)	Retrospective cohort	3013	IM P + oral DYD vs. VG P (gel) + oral DYD	60 mg /d + 10 mg×3 /d 90 mg×2 /d + 10 mg×3 /d	Day 4 or 6	LBR; P＜0.028 40.8% vs. 45%
Liu Y and Wu Y (2020)	Retrospective cohort	856	IM P + oral DYD vs. VG P (gel) + oral DYD	60 mg /d + 30 mg /d 90 mg/d + 30 mg /d	Day 2 or 3	n.s. in LBR or neonatal outcomes
Turkgeldi, et al. (2020)	Retrospective cohort	214	SC P vs. VG P (8% gel)	25 mg×2 /d 90 m×2 /d	Day 6	n.s.
Vuong, et al. (2021)	Prospective cohort study	1364	VG P(tablet) + oral DYD vs. VG P (tablet)	400 mg×2 /d + 10 mg×2 /d 400 mg×2 /d	Day 4 or 6	LBR; P = 0.042 46.3% vs. 41.3%
Pabuccu, et al. (2022)	RCT	151	oral DYD vs. VG P (gel) vs. IM P	20 mg×2 /d 90 mg×2 /d 50 mg×2 /d	5 days of P	n.s.

Note: RCT, randomized clinical trial; VG P, vaginal progesterone; IM P, intramuscular progesterone; DYD, dydrogesterone; SC P, subcutaneous progesterone; d: day; OPR, ongoing pregnancy rate; LBR, live birth rate; n.s., not significant.

Aqueous P, the most recently developed form of P preparation, allows for subcutaneous (SC) administration. SC P appears to be biologically equivalent to intramuscular (IM) P in terms of P exposure [31, 32], but does not cause as much pain as IM injections and does not cause induration or sterile abscesses. There is limited information available about SC P performance in hormone replacement therapy (HRT) cycles, despite its proven usefulness in fresh embryo transfer cycles [33, 34]. According to a retrospective cohort study [35], clinical pregnancy rates achieved with SC P (25 mg, twice daily) were comparable to those achieved with P vaginal gel (90 mg, 8%, twice daily) in the HRT cycles. Future studies must further determine whether subcutaneous P can be used as a substitute for intramuscular and vaginal routes for luteal phase support in the HRT cycles.

Except for dydrogesterone, oral P is normally avoided due to its low absorption and reduced efficacy in assisted reproductive technology (ART). Dydrogesterone is frequently used as a complement to other administration routes to improve pregnancy outcomes. When compared to vaginal micronized P alone, Vuong et al. [36] reported that vaginal micronized P plus dydrogesterone was associated with greater live birth rate (LBR) (46.3% vs. 41.3%, p = 0.042), and lower miscarriage rate (3.4% vs. 6.6%, p = 0.009). However, there was no discernible improvement in pregnancy outcomes with dydrogesterone alone for luteal phase support (LPS) [37–40].

It is also unclear if an abnormally high circulating P value generated by a non-optimal dosage and route of P, played a role in the negative obstetric result. A retrospective study [41] using vitrified cleavage-stage embryos transfer in HRT cycles compared both the LBR and neonatal outcomes in two groups consisting of vaginal gel Crinone (90 mg per day) and 60 mg per day IM P, as well as supplementation with dydrogesterone orally at 30 mg per day in both groups. They reported that a relatively higher serum P level (greater than 41.82 pmol/L at day 14 post-FET) induced by IM regimen did not increase newborn birthweight or prolong gestational weeks when compared to vaginal regimen. One of the study’s limitations was that obstetric data, such as gestational hypertension, pre-eclampsia, placenta accreta, and previa, were not available, impeding a better understanding of the impact of high serum P levels on placental development in pregnancy. To our knowledge, this is the first research to show a correlation between progesterone regimen procedures and neonatal outcomes.

In most studies, estrogen and progesterone are continued until the 12th gestational week in cases of pregnancy [13, 42], when placental autonomy is established to replace the absent corpus luteum [43]. During the luteal phase, progesterone plays an important role in implantation and maintaining a healthy pregnancy, but the role of estrogen is not clear.

In conclusion, the available evidence on the optimal progesterone preparation in HRT FET cycles is far from conclusive. The most common routes of administration are vaginal and IM, and more research on these agents and their combinations is needed to improve the live birth rate while ensuring the safety of both mother and baby. The rescue P protocol needs further exploration. As a novel technique, the safety and efficacy of subcutaneous progesterone must be further verified.

#### Duration of P exposure before transfer

The absence of corpus luteum (CL) in HRT makes the duration of P before transfer crucial to the outcomes of pregnancy in FET cycles. It is well recognized that inappropriate P duration before transfer can result in an out-of-sync endometrium and embryo, leading to early pregnancy loss [44]. However, evidence demonstrates that pregnancies can occur after very short progesterone supplementation, indicating that a short duration of P supplementation before FET is sufficient to create a receptive endometrium [45, 46]. On the other hand, a randomized controlled trial (RCT) [47] suggested that transferring a vitrified-warmed cleavage stage embryo following overnight culture on the third day of P administration caused a statistically significant increase in early pregnancy loss compared to transferring on the fifth day of progesterone administration.

**Fig. 1:**
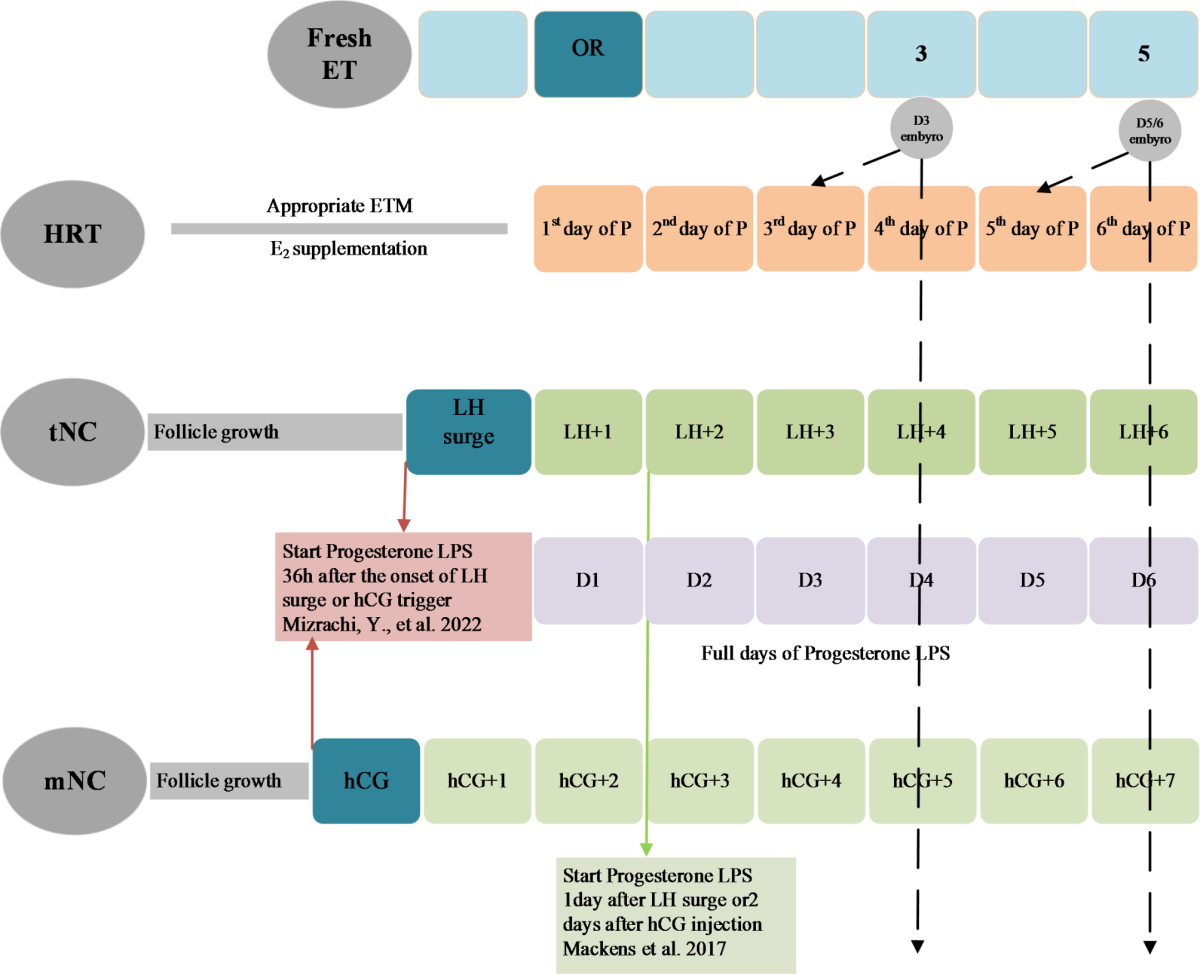
Proposal of timing of embryo transfer in HRT, tNC, and mNC and progesterone LPS in tNC and mNC. Notes: ET: embryo transfer; HRT: hormone replacement therapy; tNC: true natural cycle; mNC: modified natural cycle; ETM: endometrial thickness; E_2_: estradiol OR: oocyte retrieval; LH: luteinizing hormone; LPS: luteal phase support; hCG: human chorionic gonadotropin D: day; P: progesterone

No consensus has been reached on the optimal duration of P administration before transferring a vitrified-warmed blastocyst. However, blastocysts have been transferred from 5 to 7 days of P administration in different HRT protocols nowadays. Based on available evidence, it has been proposed that blastocysts be transferred at least on the day of “P + embryonic age” in the HRT cycle, which is typically one day after the start of P treatment [48]. A current retrospective cohort study with logit-transformed propensity score matching (PSM) [49] reported that single blastocyst transfer on the sixth day of P administration was associated with better clinical outcomes compared with the seventh day. They also conducted a more detailed subgroup analysis of the effects of blastocyst development days on pregnancy outcomes and indicated that day 5 blastocysts had significantly higher LBR and clinical pregnancy rates (CPRs) than day 6 blastocysts [49]. In contrast to this study, an absolute difference of 16% in clinical pregnancy rate was powered to detect in an RCT when FET was performed on the seventh rather than the fifth day of P administration in an HRT cycle, although the difference was not statistically significant [50]. Meanwhile, the same team’s retrospective cohort analysis [51] reported that FET on the sixth day of P treatment resulted in LBR comparable to embryo transfer on the seventh day of P administration. Additionally, they performed a subgroup analysis on the effect between the days of P administration and blastocyst development days and revealed significantly higher miscarriage rates for day 6 blastocysts transferred on the sixth day of P supplementation compared with their transfer on the seventh day. But for day 5 blastocysts, no difference was found on the sixth or seventh day of P administration. It is suggested that the day 6 blastocyst has a specific embryo-endometrium synchrony pattern with different developmental potential and a different window of implantation (WOI) compared with the day 5 blastocyst. Therefore, the optimal duration of P exposure before FET for day 5 and day 6 blastocysts may not be equal**(**Fig. 1**)**.

In conclusion, the available evidence suggests that the optimal P exposure before cleavage stage embryo transfer in hormone replacement therapy is three or four days. For the frozen-thawed blastocyst transfer cycle, we recommend the following priority order: (1) Preferentially select the blastocyst on day 5 over day 6. (2) Transfer on sixth or seventh day of P administration is preferable than the fifth day.

### GnRH-a in HRT

Although unplanned, spontaneous follicular growth and ovulation are rare conditions in HRT cycles, they occur without pituitary suppression using GnRH agonist, with incidence ranging from 1.9% [52]to 7.4% [53]. Once spontaneous follicular growth and ovulation occurs, HRT cycles might be cancelled, causing financial and time losses, as well as increasing emotional pressure on the patients.

A RCT conducted at two centers compared the reproductive outcomes of HRT-FET with or without GnRH-a pretreatment in the general infertile population [54]. It was shown that the CPR, implantation rate, LBR, early pregnancy loss rate, and ectopic pregnancy rate were all equivalent across the two protocols [54]. These findings are consistent with Cochrane meta-analysis which showed comparable clinical pregnancy and miscarriage rates following FET in women with and without GnRH-a pretreatment [55]. Although suppressing the hypothalamus-pituitary axis to avoid ovulation can be achieved by GnRH-a administration prior to HRT, HRT FET cycles without GnRH-a co-treatment seem to be more patient-friendly due to the reduction in costs and potential side effects associated with down-regulation. The use of GnRH-a for individuals with specific types of infertility is as follows:

#### Patients with adenomyosis

Adenomyosis is a benign gynecological disease in which the endometrial stroma invades the uterine myometrium. It is also an estrogen-dependent inflammatory disease. To improve reproductive outcomes, numerous protocols have been tried. GnRH-a therapy, which has a hypoestrogenic effect, may be effective in reducing inflammatory reactions and angiogenic responses, decreasing the size and demarcation of adenomyotic lesions in women with adenomyosis [56]. In a retrospective study, Niu et al. [57] compared outcomes of adenomyosis patients undergoing long-term pituitary downregulation before HRT-FET and indicated that clinical pregnancy, implantation, and ongoing pregnancy rates were significantly higher than in women not pretreated with GnRH-a. Similarly, another retrospective study including 43 FET cycles suggested that FET following 2–3 months of GnRH agonist pretreatment tended to increase the pregnancy rate in patients with adenomyosis [58]. However, a recent retrospective study [59] showed no observable effects of GnRH-a prior to HRT-FET on pregnancy outcomes of patients with adenomyosis. Most patients in this study underwent GnRH-a downregulation only once, which may have contributed to poor results in terms of GnRH-a-mediated improvements in pregnancy outcomes, as well as the fact that many patients did not experience severe adenomyosis. Additionally, GnRH-a pretreatment might not benefit all patients with mild, undiagnosed adenomyosis. A recent meta-analysis [60] suggested that downregulation could only be helpful for patients with stage III or IV endometriosis, not for those with mild endometriosis.

#### Patients with polycystic ovary syndrome (PCOS)

PCOS is a complex endocrine disorder characterized by overproduction of androgen and LH, increased inflammatory variables related to endocrine diseases, decreased expression of avβ3 integrin and glycodelin, and reduced endometrial receptivity, fertility, oocyte maturity, and embryo quality. A recent retrospective study with a large sample size showed that GnRH-a treatment before HRT-FET was associated with a lower rate of miscarriages and a higher LBR in women with PCOS [61]. These benefits of GnRH-a pretreatment for PCOS may be due, in part, to the lowering of serum LH level, serum estradiol level, and GnRH-HCG axis activity, which, in turn, inhibits endometrial inflammation and enhances the production of endometrial adhesion molecules. However, another retrospective cohort study using PSM suggested that GnRH-a + HRT-FET protocols have a similar chance of live birth with the HRT-FET among PCOS women [62]. Similarly, current RCTs suggested that GnRH-a pretreatment did not improve LBR in patients with PCOS who received HRT-FET, but significantly increased their treatment costs [63, 64]. In addition, PCOS is associated with poor pregnancy outcomes and a higher risk of pregnancy complications. However, there was only one retrospective cohort study that compared the neonatal outcomes of PCOS women undergoing HRT-FET with or without GnRH-a suppression, and it suggested that GnRH-a pretreatment was independently associated with a decreased risk of preterm birth (PTB) and an increased risk of small for gestational age infants [61]. According to Christ et al.[65], among the three main features of PCOS, hyperandrogenemia stands alone as a risk factor for PTB. GnRH-a can reduce androgen production by inhibiting the GnRH-HCG axis.

#### Patients with recurrent implantation failure (RIF)

The optimal definition of RIF and its treatment methods are still the subject of debate [66, 67]. Prospective cohort studies have indicated that FET might improve reproductive outcomes in women with RIF [68]. Because FET creates a more synchronous uterine environment and prevents the disturbed endometrial development that results from external ovarian stimulation. However, which endometrial preparation protocol is more effective in promoting implantation in RIF patients remains controversial. A previous retrospective self-control study showed that the GnRH-a HRT protocol can increase the pregnancy success rate in FET cycles of patients who have experienced RIF following IVF treatment [69]. The same result was observed in older RIF patients of 36–43 years [70]. The researchers hypothesize that the GnRH-a HRT protocol enhances implantation-related factors and promotes optimal endometrial receptivity, leading to an improved LBR. However, these findings were questioned in a retrospective cohort study showing that a GnRH-a HRT protocol does not improve the LBR for patients with RIF [71]. Since there are no large RCTs focus on the effect of GnRH-a HRT in RIF patients, more research is required to confirm this item.

In conclusion, based on the available data, GnRH-a HRT cannot improve pregnancy outcomes in general infertile individuals while increasing the cost and duration of therapy. More RCTs are required to confirm its efficacy for RIF and PCOS patients. However, patients with severe adenomyosis may benefit from GnRH-a pretreatment that is more persistent in duration.

### Natural cycle (NC)

In a natural FET cycle, endometrial maturation is dependent on endogenous estradiol and progesterone, which are produced by the growth of a dominant follicle and stimulating the growth of the endometrium. This protocol avoids high dose of exogenous hormones, making it simpler and more physiological than other methods. Women with regular menstrual cycles can plan a natural FET in true natural cycles (tNC), with timing based on monitoring the naturally occurring LH peak and ovulation, or in hCG-triggered modified natural cycles (mNC).

#### True natural cycles (tNC)

Pinpointing the day of ovulation is crucial for timing FET in a tNC to maximize the live birth rate. The current practice is mixed and relies on LH surge documentation by daily/frequent endocrine monitoring, including serum LH, estradiol, and progesterone, combined or not with serial ultrasound assessments to confirm ovulation.

During a tNC, the rise of estradiol, originating from the dominant follicle and exceeding 200–300 pg/ml for a minimum of 50 h, triggers the LH surge. Although prior research has shown that serum P levels rise 12 h before the start of the LH surge, this increase was not thought to play a significant role in the physiology of ovulation. However, there has been renewed focus on the LH-independent increase in circulating P, manifested by a sharp increase in serum P to 0.5 ng/ml, as the trigger for the LH surge [72].

Ovulation usually occurs 24–56 h after the spontaneous LH surge. Considering the day of the LH surge as Day 0, the usual practice to perform tNC-FET at the cleavage and blastocyst stages is LH surge + 4 days and LH surge + 6 days, respectively [48]. However, there is no consensus on the definition of the LH surge. Importantly, there is a paucity of data concerning reproductive outcomes when employing different criteria for timing of FET in tNC. A recent prospective study [73] suggested that vitrified–warmed embryo transfer on follicular collapse + 5 days is equivalent to LH surge + 7/+8 and even + 9 days in a significant proportion of tNC with comparable reproductive outcomes, reflecting the high degree of flexibility of the window of implantation. However, the sample size was modest, and more research on the definition of LH surge is needed.

A urine LH test is a low-cost and convenient alternative to venipuncture for determining whether to proceed with FET. However, due to the longer urine clearance of LH, a temporal delay should be considered when comparing urinary testing to serum testing [74]. A urine LH test is positive 12–36 h after the plasma LH surge [75]. The difficulty with urine LH testing is that physiological variability makes it impossible to pinpoint the exact moment of ovulation and, as a result, the onset of the WOI. However, FET outcomes were not significantly different when performed over a range of 3 days [76, 77].

In contrast, documentation of ovulation by ultrasound is a highly reliable direct measurement. Follicular collapse is the most predictive sign of ovulation [78]. During natural cycle FET, it is not common practice to time the transfer, and when a luteinized unruptured follicle (LUF) is recorded instead of follicular rupture, pinpointing the exact transfer date becomes problematic. Lower levels of progesterone in the middle of the luteal phase and a shorter luteal phase duration have been linked to impaired luteal function in LUF cycles [79]. As a result, LUF might negatively affect embryo implantation or ongoing pregnancy in natural cycle FET. However, evidence of the impact of LUF on clinical outcomes of FET is lacking. Wang et al. [80] studied the effect of LUF on pregnancy outcomes of frozen/thawed cleavage embryo transfer and reported that LUF had no effect on FET clinical results. Note that the study used a slow freezing and rapid thawing method, therefore, the results cannot be generalized to the more common vitrification method of embryo cryopreservation [80]. A current retrospective cohort study [81] reported that LUF had a detrimental effect on pregnancy outcomes in natural cycle FET of blastocysts, especially when the LH surge level was insufficient. These findings need to be confirmed by larger sample size studies or prospective randomized trials, and a deeper understanding of the mechanism behind LUF is necessary.

#### Modified natural cycle(mNC)

To our knowledge, tNC requires strict monitoring of hormonal and follicular development. For a variety of reasons, mNC is considered more patient-friendly than tNC. For starters, there is no need for continuous hormone monitoring. In other words, after the hCG trigger is administered, the date of embryo transfer can be calculated. Furthermore, hCG injection not only induces ovulation but also promotes the luteal phase.

In mNC, ovulation is triggered with hCG when the leading follicle is 16–20 mm in diameter. According to several recent studies, focusing solely on follicular size may result in the administration of hCG either too early or too late, thereby affecting endometrial receptivity. In some cases, an early endogenous LH peak might promote premature luteinization of the endometrium, resulting in asynchrony between the endometrial and the transferred blastocyst. As a result, routine LH testing prior to hCG triggering ovulation was recommended. According to a pilot study [82], LH elevation of 13 mIU/ml prior to hCG administration may reduce clinical pregnancy rates in mNC for single euploid blastocyst transfer. However, a retrospective study of 1168 mNC-FET cycles revealed contradictory results [83]. In this clinical trial, hCG can be given at any time between the start of the LH rise (15 IU/L) and the peak level of LH (40 IU/L) without affecting the clinical outcome [83]. In any case, the research on this issue were most retrospective studies. As a result, more study is required to determine if the LH surge before hCG treatment influences pregnancy outcomes in mNC.

A recent review based on retrospective studies suggested both warming and transfer of blastocysts on hCG trigger + 7 days in mNC-FET [48]. A recent retrospective analysis indicated that women who had a spontaneous LH surge and transferred 6 days later had identical pregnancy outcomes to women who did not have a detectable LH surge on the day of the hCG trigger and transferred 7 days later [84]. They adjusted the mNC-FET time depending on LH levels of 20 mIU/ml [84]. So far, it is unclear if the timing of mNC-FET should be changed based on the LH surge, and further randomized clinical trials are desperately needed.

#### Luteal Phase support (LPS) in NC

Progesterone administration is required in HRT cycles because no endogenous P is produced, but nothing is known about the effect of LPS in true or modified natural FET cycles [48, 85].**(**Table 2**)** Luteal phase defect is thought to be uncommon in cycles involving spontaneous ovulation because the resulting corpus luteum is thought to provide all that is needed for embryo implantation [86]. However, some women undergoing fertility treatment may not produce enough progesterone. In other cases of embryo-endometrial asynchrony caused by normal physiological fluctuations or misinterpretation of laboratory results, administration of LPS may also be helpful. It is controversial to provide LPS after tNC-FET or mNC-FET, despitehat there is no physiological basis for it.

**Table 2:** LPS following NC-FET: characteristics of the included studies

Study	Design	Sample size	Method of embryo freezing	HCG ovulation trigger	LPS regime	LPS time	Primary outcomes
Lee et al. (2013)	Retrospective cohort	408	Not reported	No	IM 1500 IU uhCG	On FET day and 6 days after FET	n.s. in CPR
Lee et al. (2017)	RCT	450	Not reported	No	IM 1500 IU uhCG	On FET day and 6 days after FET	n.s. in OPR
Bjuresten et al. (2011)	RCT	435	Not reported	No	Vaginal micronized progesterone, 400 mg twice a day	Starting three days after the LH surge	LBR, P = 0.027 n.s. in CPR, MR
Wanggren et al. (2022)	RCT	488	Slow freezing/ Vitrification	No	Vaginal progesterone tablets,100 mg twice daily	Starting on the day of FET and continued for six full weeks corresponding to 8 weeks of pregnancy	LBR, P = 0.017
Kyrou et al. (2010)	Retrospective cohort	452	Slow freezing	Yes	Vaginal micronized progesterone, 200 mg three times daily	Starting one day following hCG trigger until 7 weeks of gestation	n.s. in OPR
Kim et al. (2014)	Retrospective cohort	228	Vitrification	Yes	Vaginal progesterone gel once daily	Starting 2 days after hCG trigger until 11–12 weeks of gestation	LBR, P = 0.041 MR, P = 0.044
Eftekhar et al. (2013)	RCT	103	Vitrification	Yes	IM progesterone 50 mg twice daily	Starting 36 h following hCG trigger until 10 weeks of gestation	n.s. in CPR, IR
Schwartz et al. (2019)	Retrospective cohort	231	Vitrification	Yes	Vaginal micronized progesterone, 200 mg twice daily	Starting two days following hCG trigger until 9 weeks of gestation	CPR, P = 0.020
Horowitz (2020)	RCT	59	Slow freezing/ Vitrification	Yes	Vaginal micronized progesterone, 100 mg twice daily	Starting two days following hCG trigger until 8 weeks of gestation	n.s. in CPR

Notes: LPS: luteal phase support; IM: intramuscular injection; FET: frozen–thawed embryo transfer; CPR, clinical pregnant rate; RCT: randomized controlled trial; OPR: ongoing pregnant rate; LH: luteinizing hormone; LBR: live birth rate; MR: miscarriage rate; uhCG: urine human chorionic gonadotropin ;hCG: human chorionic gonadotropin; IR: implantation rate, n.s., not significant.

Only two studies examined the use of hCG as the sole regimen for LPS in NC-FET [87, 88]. These two investigations by the same group indicated that LPS with hCG in tNC-FET did not raise the CPR: a retrospective cohort analysis and a placebo-controlled randomized clinical trial in which women in the LPS group received two doses of 1500 IU of urinary hCG on the day of FET and 6 days later [87, 88].

Progesterone supplementation in tNC-FET boosted the number of live births following frozen-thawed embryo transfer, according to a recent prospective RCT [89]. As a result, they suggested that it should be taken into clinical practice [89]. The results were consistent with those of a prior large RCT [90]. But the evidence for progesterone supplementation for mNC-FET is contradictory, being either in favor of progesterone LPS [91, 92] or showing no significant differences in terms of outcomes [93–95]. Moreover, two recent meta-analyses indicated that progesterone supplementation after NC-FET improves LBR [96, 97]. However, more research is needed to determine the best form and dose of progesterone for administration during the luteal phase of NC-FET.

Starting LPS too early or too late could result in asynchrony between the endometrium and embryo stages of development, resulting in poor embryo implantation. There have been no studies focus on the best timing of P administration in natural FET cycles, and clinical practice varies widely across the globe. Mackens et al. [48] proposed starting LPS 1 day after LH surge in tNC-FET or 2 days after HCG injection in mNC-FET, based on the notion that the endometrium requires 6 days of post-ovulatory P to allow blastocyst implantation. According to a recent review [98] based on the premise that P supplementation needs to closely mimic the natural pattern of P secretion by the corpus luteum, progesterone support in natural FET cycles should begin 36 h after the serum LH surge measured in the morning, or 36 h after HCG. They also recommended starting P supplementation 24 h following a positive urine LH test [98]. To date, no randomized trial has studied the timing of the onset of LPS in the natural cycle. **(**Fig. 1**)**

In conclusion, P supplementation was associated with a higher LBR and CPR in tNC-FET cycles. However, the effectiveness of P supplementation in mNC-FET cycles should be further verified by larger RCTs. Using hCG for LPS in tNC-FET is not supported by evidence. The above suggestions are speculation based on the available evidence, and further RCTs are urgently needed to determine how or when exactly LPS should be administered during natural FET cycles.

### Time to go “natural”?

HRT cycles are more flexible and convenient than natural cycles, for their lower cancellation rate and they are easy to schedule transplants. The HRT protocols are currently performed at the discretion and preference of the treating physician. However, for women capable of ovulation, the HRT FET regimen cause a strong deviation from physiology [99]. Trials aimed at optimizing HRT FET cycle regimens for ovulatory women are therefore no longer encouraged, especially considering the safety of women and fetus. Recent prospective and retrospective cohort studies have consistently shown a considerably increased risk for pre-eclampsia in HRT FET cycles compared to ovulatory FET cycles [100–104]. This may be caused by the absence of a CL and its products in early pregnancy, as well as the negative consequences of exposure to exogenous hormones, which may link to an increased risk of thromboembolic events and placentation deficiency [105, 106]. For this reason, women capable of ovulation undergoing FET are proposed to prioritize natural cycle protocol. Optimizing FET under the circumstances of an ovulatory cycle whenever feasible includes different types of monitoring and different types of luteal and early pregnancy support for ovulatory women [107].

### Endocrine monitoring

#### Monitoring during estrogen supplementation in HRT

In addition to checking the endometrial thickness via ultrasonography prior to FET, the necessity of endocrine monitoring in artificial cycles remains controversial. Serum estradiol levels during the proliferative phase in artificial cycles for FET may affect endometrial receptivity. A large retrospective cohort study (n = 3857) [108] that investigated the effects of serum estradiol levels before P administration on FET outcomes reported a statistically significant negative association between higher estradiol levels (≥ 400 pg/ml) and both lower LBR and CPR. However, the ideal serum estradiol threshold before progesterone treatment remains unclear. In contrast to these results, Mackens et al. [109] suggested no significant correlation between blood estradiol levels prior to P administration and LBR in a large retrospective study of 1222 HRT FET cycles.

During the estrogen-only phase of HRT cycles, the endometrium thickens without follicle development. However, sometimes there is a rise in serum LH. Griesinger et al. [110] reported no significant correlation between serum LH levels and the likelihood of clinical pregnancy in a prospective study of 513 HRT FET cycles. However, according to another recent retrospective cohort study [111], low serum LH levels before P initiation in HRT FET cycles of ovulatory women were negatively associated with LBR. Although it is not a key predictor, monitoring serum LH levels before P initiation may help to obtain the best clinical outcomes for scheduling embryo transfer.

#### Monitoring during luteal phase in HRT

Circulating progesterone level may be associated with treatment success in FET. Serum progesterone levels peak during the WOI and have been used as a marker of endometrial receptivity in natural conception and ART treatment [112]. The purpose of monitoring serum progesterone during the difference stages of the luteal phase may vary. The purpose of endocrine monitoring before embryo transfer is to evaluate endometrial P, and to perform rescue P protocol in case of lower serum P level [113].

Low blood P levels (8.8–10.6 ng/ml) through the vaginal route around the time of the frozen blastocyst transfer have been linked to a lower LBR [114–119]. Variations in progesterone cut-off levels may be related to differences in study populations, different immunoassays for serum progesterone measurements [120], different days of serum progesterone measurements [119], or different progesterone dosages [114, 115, 121, 122]. Regardless, the bottom P threshold associated with pregnancy outcomes appears around 10 ng/ml [123], which is close to the value reported as an adequate level of P production by the CL in the mid-luteal phase of natural cycles [124, 125], which is essential for endometrial secretory transformation in preparation for embryo implantation. In contrast, the P cutoff values reported for HRT cycles using IM progesterone are higher, reaching 13.6 ng/ml [126]or even 20 ng/ml [127]. This may be explained by progesterone’s pharmacokinetics and pharmacodynamics among delivery methods. The rescue P supplement according to the P level before implantation needs to be intensively explored in the near future as there are still controversy and discrepancies in various studies. Additionally, it is important be noted that the serum P could not accurately reflect the real impact when dydrogesterone is used in LPS.

Despite being reported in three studies, it remains uncertain whether there is a ceiling threshold of serum P in HRT cycles, and whether exceeding the threshold will negatively impact reproductive outcomes [122, 128, 129]. In one retrospective study conducted by Kofinas et al., which included 213 patients undergoing euploid blastocyst transfer and HRT with IM progesterone, a ceiling effect was observed with serum progesterone levels exceeding 20 ng/mL on the sixth day of progesterone administration [129]. They observed that progesterone levels > 20 ng/ml on the day of transfer were associated with a decreased ongoing pregnancy rate and LBR [129]. Yovich et al. reported an optimal serum progesterone range of 22.01– 31.1 ng/ml on the sixth day of progesterone initiation [128]. Patients with serum progesterone levels exceeding 31.1 ng/ml had lower implantation rates and LBR [128]. In another recent prospective cohort study, a ceiling effect was noted for CPR and LBR with serum progesterone levels ≥ 32.5 ng/ml [122]. Prospective studies with large sample size are needed to elucidate the exist of serum ceiling P level.

Once pregnancy is established, it appears that the higher the progesterone concentration, the more likely the pregnancy continues. A recent prospective cohort study [130] sought to evaluate potential differences in serum P levels throughout the late luteal phase (days 4, 7, and 11 after ET) based on pregnancy outcomes and reported that patients with an ongoing pregnancy had higher levels of serum P than the rest, particularly those with a negative result or pregnancy loss. And there was an increasing trend of serum P level throughout the luteal phase days. Consequently, serum progesterone levels throughout the late luteal phase may help us predict an ongoing pregnancy or an implantation failure, but a serum progesterone threshold that could predict successful or failed implantation has not been set.

In conclusion, serum progesterone monitoring has an important role not only during implantation but also in pregnancy maintenance. It is recommended to check serum P levels on or before ET day, but it is yet unclear if P monitoring is essential at any other points. Future research needs to validate the luteal serum concentration of P required to ensure an optimal endocrine milieu during embryo implantation and early pregnancy maintenance after FET treatment.

#### Monitoring during tNC

To accurately prepare for FET in tNC, the timing of spontaneous ovulation must be determined. This requires close endocrine and transvaginal ultrasonographic monitoring. Both abnormal estrogen and progesterone levels lead to a spectrum of alterations in the endometrium, such as changes in histological features as well as gene and cytokine expression related to endometrial receptivity. However, the role of endocrine monitoring in tNC is debatable. Many patients with normal menstrual cycles were observed to have delayed ovulation during monitoring; however, the length of the follicular phase in tNC-FET did not appear to affect pregnancy rates or LBRs. But when the dominant follicle develops with an accelerated trajectory, reflected by an elevated estradiol level (> 100 pg/ml) to surge ≤ 4 days, pregnancy rates or LBRs decrease [131]. In addition, a previous, smaller retrospective study also reported a high incidence (28.4%) of progesterone elevation of 5 nmol/L or more before the LH surge in patients undergoing tNC-FET [132]. Overall, no differences in CPR and OPR were observed between patients with and without elevated progesterone [132]. A subgroup analysis within that study suggested it was not the level but the duration of progesterone exposure before the LH surge that was responsible for lower pregnancy rates [132].However, progesterone values below a specific level in the luteal phase may be associated with impaired pregnancy outcomes in NC-FET. The significance of serum progesterone levels before the ET day of tNC, however, has only been examined in one retrospective cohort study [124]. It reported that normal ovulatory women undergoing tNC-FET with serum progesterone levels < 10 ng/mL on the day before blastocyst transfer have a considerably lower LBR than those with higher levels [124].

#### Monitoring during mNC

When it comes to mNC-FET cycles, endocrine monitoring is controversial. The clinical value of monitoring serum LH levels before HCG trigger has been discussed previously, but few studies have explored the influence of serum progesterone levels on the hCG day on the clinical outcomes in mNC-FET cycles. In a recent retrospective study, the threshold effect analysis of the serum P level on the day of hCG showed that the LBR decreased significantly when the P level reached or exceeded 1 ng/mL in mNC-FET cycles [133]. The impact of endocrine level of the luteal phase in mNC-FET has not yet been investigated.

### Endometrial Assessment

Endometrial receptivity is considered a crucial factor for the success of FET. Ultrasonography is a convenient and non-invasive method routinely used to check the endometrium. Several sonographic parameters have been developed to identify endometrial receptivity, among which the endometrial thickness (EMT) and the endometrial pattern are widely accepted as prognostic indicators [134–137]. A recent retrospective study of 20,114 FET cycles reported that an endometrial thickness of 7 mm before embryo transfer reduced clinical pregnancy rates [134]. Others have observed an upper limit of EMT beyond which the implantation rate also decline, with this limit being larger than 13 mm in an observational cohort study [135]. This shows that there is an optimum range before embryo transfer, rather than just a minimal threshold EMT. Recently, our data manifested that both the thin and thick endometria were associated with an increased risk of hypertensive disorders of pregnancy in FET cycles [138], and clinicians should focus on adjusting EMT especially in the risk population.

More recently, some studies focused on the endometrial compaction during the early luteal phase. The endometrial compaction defined as a decrease in EMT between the end of the estrogen-only phase and the day of embryo transfer. The current literature on endometrial compaction is highly heterogeneous. Two studies by the same group [139, 140], who retrospectively analyzed embryo transfer images taken by abdominal ultrasonography (AUS) in HRT cycles, observed higher ongoing pregnancy rates in FET cycles that reported endometrial compaction of ≥ 5% compared with cycles in which the endometrium neither compacted nor expanded. This was supported by Youngster, M., et al. [141] in a retrospective observational study. However, several recent studies, including HRT cycles alone [142, 143] or in combination with modified natural cycle (mNC) [144–146], failed to link endometrial compaction of ≥ 5% with pregnancy rates or live birth rates (LBRs). To avoid errors introduced by ultrasound measurement, EMT in these studies [142, 143] was assessed via both transvaginal ultrasonography (TVUS) and AUS in all patients on the day of embryo transfer. Furthermore, estrogen and progesterone concentrations were accessed to investigate hormonal reasons behind this event, and no connection with endometrial compaction was discovered. However, progesterone receptor deficiency or resistance may explain the difference of endometrial compaction among cycles.

In conclusion, based on the current data, examining the endometrium can establish the viability of an embryo transfer and may help clinicians adjusting EMT for improving perinatal outcomes. But there seems to be uncertainty benefit from evaluating endometrial compaction during HRT-FET or mNC-FET.

## Conclusion

Although the use of FET has increased globally, research on the ideal endometrial preparation strategy for different population is ongoing. As FET protocols have been established, more attention has been paid to the details of the treatment process to improve the outcome of FET. Specific items of focus include the timing of ovulation in natural cycle, as well as the ideal progesterone supplement during HRT-FET cycles. Maternal, obstetrical, and neonatal outcomes, in addition to LBR, should be considered in evaluating these specifics.

In HRT cycles, evidence suggests that estrogen with oral or transdermal routes has similar reproductive outcomes, but transdermal route yields more steady state levels of estradiol and could be preferred for induction of endometrial receptivity. Clinicians can be flexible in scheduling estrogen supplement procedure between 7 and 36 days before P administration on account of maintaining optimal pregnancy rates and mother-infant safety. There is a paucity of data on the impact of different routes of progesterone administration on perinatal outcomes in HRT cycles. Although vaginal and intramuscular routes of administration are prevalent, more research on these agents and their combinations is required to enhance LBR while ensuring maternal and neonatal safety. The safety and efficacy of subcutaneous progesterone as a novel technique must also be verified further. In term of proper duration of P exposure before transfer, the available evidence suggests that vitrified blastocyst transfer is prefer on sixth or seventh day of P administration than the fifth day.

Currently, low-quality evidence points toward the NC (tNC/mNC) being superior to HRT, but largely retrospective data had provided evidence for the superior of NC over the HRT protocol, specifically increased rates of hypertensive disorders of pregnancy in HRT cycles. Although the debate on whether clinicians should prescribe HRT cycles in ovulatory patients continues, the NC protocol should be the priority choice when ovulatory women undergoing their first FET treatment. However, prediction of the optimal time for embryo transfer is the key issue for NC protocol, even though the WOI is relative wide. Likewise, the hormonal support protocol in HRT is still the focus of concern and lack of consensus on guidelines. So, high quality studies are necessary to identify the cutoff values and clinical relevance of the detail parameters and the procedures, which will help to provide individualized endometrial preparation for both pregnancy and prenatal outcomes improvement.

## Data Availability

Not applicable.

## Abbreviations

ART: assisted reproductive technology FET:frozen–thawed embryo transfer
OHSS: ovarian hyperstimulation syndrome
LBR: live birth rate
hCG: human chorionic gonadotrophin
HRT: hormone replacement therapy
GnRH-a: gonadotropin-releasing hormone agonist
ET: embryo transfer
IM: intramuscular
SC: subcutaneous
RCT: randomized controlled study
CL: corpus luteum
PSM: propensity score matching
WOI: window of implantation clinical pregnancy rate
PCOS: Polycystic Ovary Syndrome
LH: luteinizing hormone
PTB: preterm birth
RIF: recurrent implantation failure
IVF: in-vitro fertilization
NC: natural cycle
tNC: true natural cycl
LUF: luteinized unruptured follicle
mNC: modified natural cycle
LPS: luteal phase support
EMT: endometrial thickness
